# Early Graft Loss after Kidney Transplantation: Endothelial Dysfunction of Renal Microvasculature

**DOI:** 10.1155/2018/4074209

**Published:** 2018-07-25

**Authors:** N. Kojc, M. Perše, J. Pleško, Ž. Večerić-Haler

**Affiliations:** ^1^Institute of Pathology, Faculty of Medicine, University of Ljubljana, 1000 Ljubljana, Slovenia; ^2^Institute of Pathology, Medical Experimental Centre, Faculty of Medicine, University of Ljubljana, 1000 Ljubljana, Slovenia; ^3^Department of Nephrology, University Medical Centre Ljubljana, 1000 Ljubljana, Slovenia

## Abstract

Decision process about the acceptance of the deceased donor kidney for transplantation might be challenging. Although histological evaluation of pretransplant donor kidney biopsy provides reliable information regarding cortical necrosis, vascular thrombosis, extensive global glomerulosclerosis, and interstitial fibrosis/tubular atrophy, only electron microscopy enables thorough and reliable insights into microvasculature changes of kidney graft. The aim of the present paper is to briefly present two cases of early kidney graft loss. In one case, the donor was exposed to long-term extracorporeal membrane oxygenation (ECMO); in the other case, the donor experienced Takotsubo cardiomyopathy. In both cases, light microscopy of pretransplant biopsy found no pathology or significant discrepancy in morphology of kidney graft, while electron microscopy revealed severe endothelial dysfunction of renal microvasculature. Our results suggest that severe injury of renal microvasculature with relatively preserved tubular epithelium may be associated with some conditions of deceased kidney donors leading to early kidney graft nonfunction and loss. Further studies are needed to determine prognostic significance of severe ultrastructural microvasculature lesions and to evaluate disease states and conditions that could be associated with severe endothelial dysfunction of kidney graft.

## 1. Introduction

Since 1950s, when the first renal transplantation was performed, continuous progress in the replacement therapy has resulted in improved kidney transplant outcomes. Long waiting lists for renal transplants and shortage of living and standard criteria deceased donors have led to the expansion of donor pool, involving an increasing number of suboptimal and marginal donors [1]. Studies have shown that the use of kidneys from expanded criteria deceased donors can be associated with worse long-term graft outcomes in some cases and may increase the risk of early graft loss [2, 3]. A few retrospective and prospective studies have examined various clinical and histological parameters for their usability to predict the performance of kidneys after transplantation, but none of them are standardized or widely applied [1, 4–8]. Therefore, the decision process to accept or reject suboptimal kidney is complex and challenging.

To help judge the quality and to avoid unacceptably high discard rates of deceased donor organs, pretransplant kidney biopsy is recommended [9]. In our center, pretransplant kidney biopsies are routinely performed. However, due to the time consuming procedures needed for preparation of permanent tissue samples for light and electron microscopy, results are usually available after transplantation. In urgent cases, when clinicians are faced with difficult decision and biopsy evaluation is needed before a graft is accepted for transplantation, histologic analysis is performed on frozen donor kidney sections.

According to our experience, both light and electron microscopies of pretransplant biopsy offer valuable information on graft quality. Light microscopy offers an insight into organ pathology, including acute cortical necrosis, acute tubular injury, arterial/ arteriolar thrombosis, and chronic lesions (like percentage of glomerulosclerosis, interstitial fibrosis and tubular atrophy, and arteriolosclerosis), while electron microscopy provides insights into the ultrastructural changes that cannot be appropriately evaluated by light microscopy, such as basement membranes, microvasculature endothelium (endothelial cells in glomeruli, peritubular capillaries, and arteriole/small arteries), or cellular structures of renal epithelium. Such assessment (morphological and ultrastructural) of a kidney graft enables precise evaluation of the quality of the kidney graft and accurate differentiation of the preexisting changes from those arising after transplantation. In addition, it enables critical evaluation of observed ultrastructural alterations of the kidney graft in cases of early graft loss.

Recently, we were faced with two cases of early graft loss. Although histologic evaluations of pretransplant biopsies were promising in both cases, subsequent electron microscopy revealed severe endothelial dysfunction of renal microvasculature. It is tempting to speculate that the injury of renal microvasculature on the pretransplant renal biopsy might be associated with early kidney graft nonfunction and loss. Therefore, the aim of the present paper is to demonstrate our recent findings and to briefly discuss potential risk factors associated with endothelial dysfunction of exposed kidney grafts.

## 2. Endothelial Dysfunction of Renal Microvasculature and Early Graft Loss

Endothelial dysfunction, more appropriately considered as endothelial activation, represents a switch from a quiescent phenotype toward phenotype showing host defense response. Endothelial cells can lose integrity, progress to senescence, and detach into the circulation [10].

On transmission electron microscopy, activated glomerular endothelial cells are swollen, with extensive microvillus transformation of endothelial cell membrane, and loss of fenestration. In severe injury, endothelial cells may rupture leading to loss of intracellular content/debris into capillary lumen followed by detaching and loss of endothelial cells (personal observation).

To get an insight into the endothelial cell alterations a blinded light and electron microscopy analysis was performed on 50 consecutive pretransplant biopsies obtained from deceased donors after brain death. Tubular injury was assessed according to preservation of tubular epithelial cells, preservation of apical brush border evaluated in periodic acid Schiff (PAS) stain, and estimated percent of vacuolization in tubular epithelial cells.

**Figure 1:**
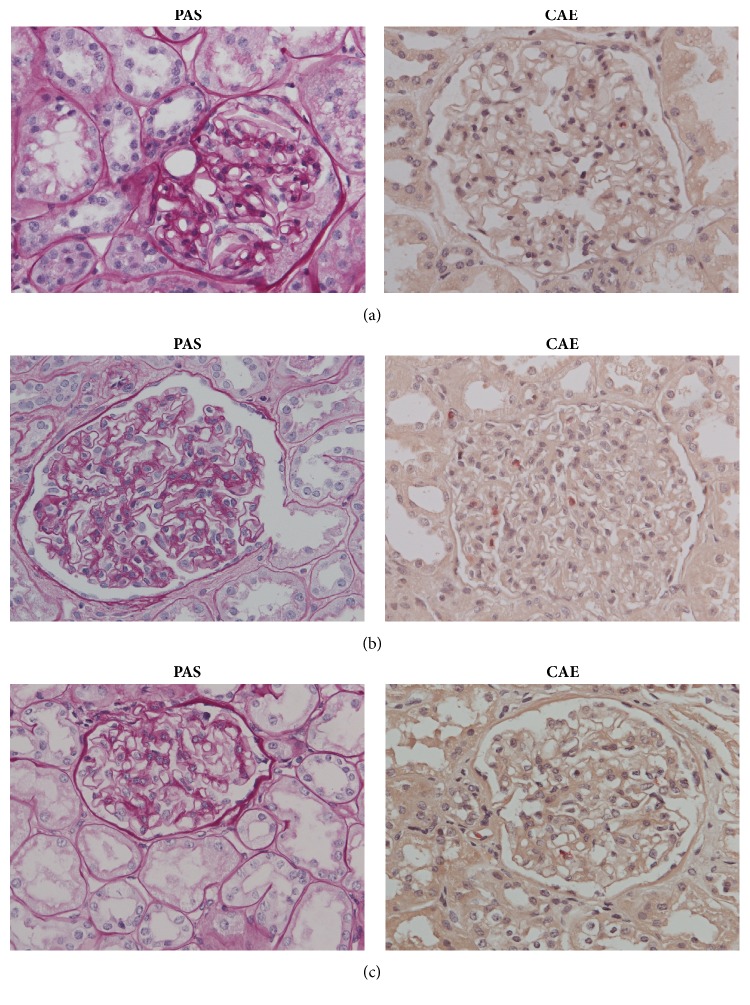
**Light microscopy of kidney graft biopsy: **magnification 400x; periodic acid Schiff (PAS) stain; chloroacetate esterase (CAE) stain.** (a) Biopsy from average deceased donor.** PAS: unremarkable glomerulus and mild acute tubular injury: proximal tubules show epithelial attenuation and focal vacuolization. The lumens appear dilated due to thinning of apical cytoplasm and focal loss of apical brush borders. CAE: in some glomeruli, there is up to one neutrophil (0-1, average 0.4).** (b) Biopsy from deceased donor exposed to ECMO.** PAS: mild acute tubular injury, similar to average preimplantation biopsy. Light microscopy showed unremarkable glomerulus with some neutrophil granulocytes. CAE: special stain revealed intraglomerular neutrophils, up to 6/ glomerulus (1-6, average 3). Sparse neutrophils are also in peritubular capillaries. In small artery above glomerulus, endothelial cells appear shrunken and detached.** (c) Biopsy from deceased donor exposed to Takatsubo cardiomyopathy.** PAS: mild acute tubular injury, similar to average preimplantation biopsy. Light microscopy shows unremarkable glomerulus. CAE: special stain revealed intraglomerular neutrophils, up to 2/ glomerulus (0-2, average 0.8). Sparse neutrophils are also in arterioles and peritubular capillaries.

By light microscopy, glomeruli in all biopsies appeared unremarkable (Figure 1(a)-PAS). Chloroacetate esterase (CAE) stain revealed up to one neutrophil (0-1, average 0.4) in glomeruli of 48/50 biopsies (Figure 1(a)-CAE). None of pretransplant biopsies showed glomerular or vascular thrombotic microangiopathy. In two biopsies, light microscopy showed unremarkable glomeruli with increased intraglomerular neutrophils, up to 6 per glomerulus (1-6, average 3) in the first (Figure 1(b)-PAS, CAE) and up to 2 per glomerulus (0-2, average 0.8) in the second case (Figure 1(c)-PAS, CAE). All examined biopsies revealed similar mild to moderate acute tubular injury without significant deviations (Figures 1(a), 1(b), and 1(c)-PAS).

**Figure 2:**
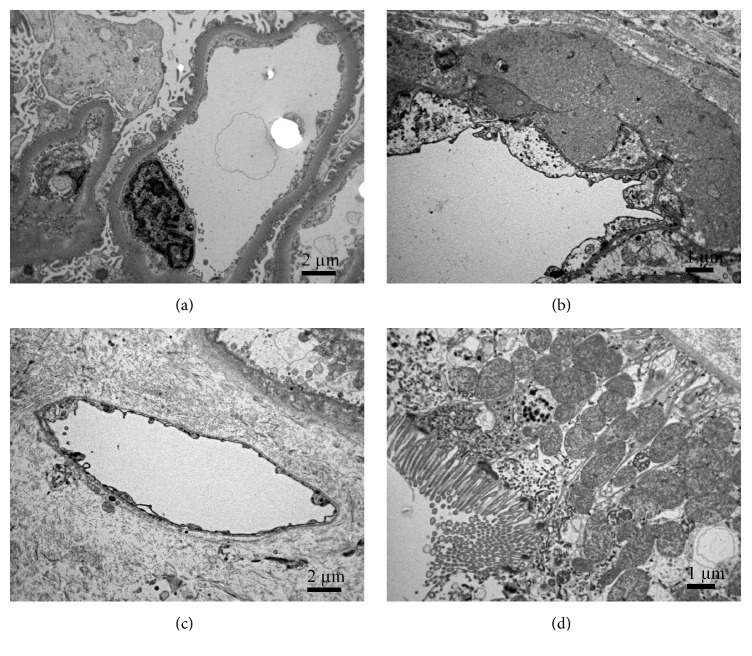
**Ultrastructure of average preimplantation kidney biopsy from deceased donor. (a)** Normal endothelial cell with fenestrated endothelium covers glomerular capillary lumen. Cytoplasmic membrane of the endothelial cell forms scattered endothelial projections (microvilli).** (b)** The normal endothelial cells in arteriole. Beneath endothelial cells, there is transmural hyalinosis indicating arterial hypertensive disease in the donor.** (c)** Normal endothelium in peritubular capillaries.** (d)** Slightly attenuated but preserved tubular epithelial cells with desmosomes. Apical brush border is slightly thinner.

Interestingly, ultrastructural examination showed similar features in 48/50 cases. Glomerular endothelial cells showed preserved fenestration with scattered endothelial projections (microvillus transformation of cell membrane). Focal mild endothelial cell swelling was observed in some cases with no or little intracellular debris in glomerular capillary lumens and no detached endothelial cells (Figure 2(a)). Endothelial cells in arterioles/small arteries appeared unremarkable or slightly swollen (Figure 2(b)). Peritubular capillaries had intact monolayer of basement membrane and were covered with normal delicate endothelial cells without swelling or detachment (Figure 2(c)). Tubular epithelial cells were attenuated with thinning of apical brush border, but showing intact desmosomes (Figure 2(d)). In all 48 cases there was no graft loss in 6-month follow-up.

**Figure 3:**
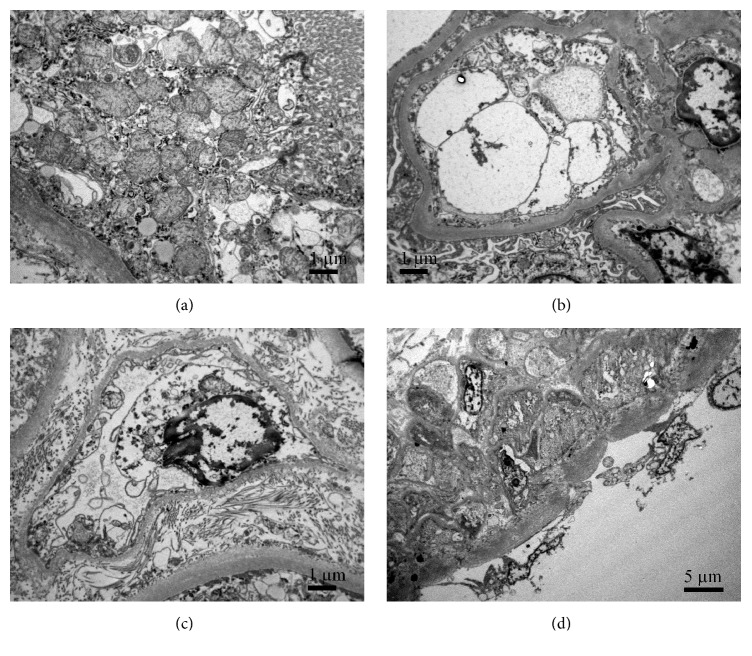
**Ultrastructure of preimplantation kidney biopsy from deceased donor exposed to ECMO. (a)** Tubular epithelial cells are attenuated but preserved showing intact desmosomes. Apical brush border is slightly attenuated, similar to all other deceased kidney biopsies.** (b)** Glomerular endothelial cells are partly detached from glomerular basement membrane, swollen and disintegrated. Swollen endothelial cell, extracellular debris, and numerous microvilli fill the lumen of glomerular capillaries.** (c)** Endothelial cells in peritubular capillary are swollen and the cytoplasmic membrane forms endothelial projections (microvilli).** (d)** Endothelial cells in small artery are completely detached and shrunken.

**Figure 4:**
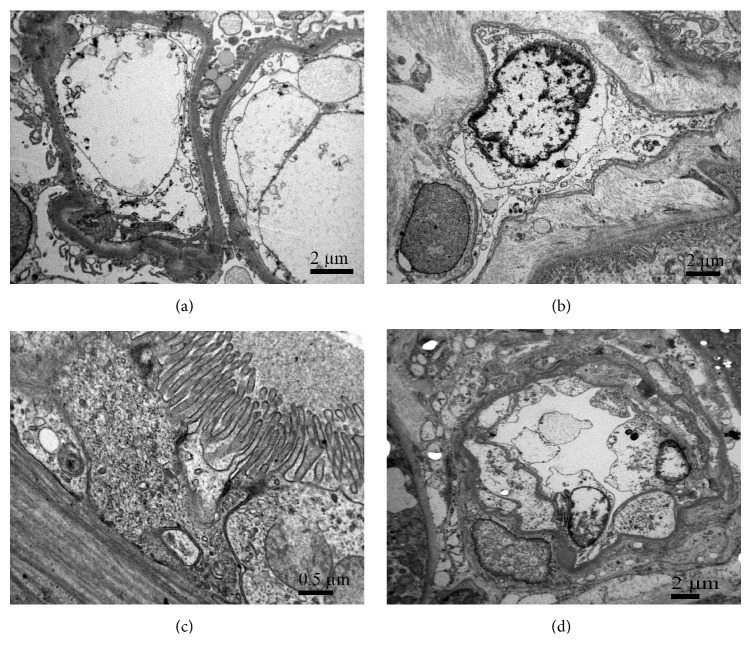
**Ultrastructure of preimplantation kidney biopsy from deceased donor exposed to Takotsubo cardiomyopathy (TCM). (a)** Glomerular endothelial cells are swollen, disintegrated, and partly detached from glomerular basement membrane. Swollen endothelial cell and extracellular debris fill the lumen of glomerular capillary.** (b)** Swollen endothelial cell in peritubular capillary. Their** c**ytoplasmic membrane forms endothelial projections (microvilli).** (c)** Tubular epithelial cells are preserved but attenuated. Desmosomes are clearly seen. Apical brush border is thinner.** (d)** Arteriole with swollen and partly detached endothelial cells.

In contrast, 2/50 cases with increased intraglomerular neutrophil granulocytes showed significant and severe ultrastructural changes of renal microvasculature without significant changes in tubular epithelium (Figures 3(a) and 4(c)).

Changes in glomerular endothelial cells looked similar in both cases (Figures 3(b) and 4(a)). There were loss of fenestration and extensive swelling of glomerular endothelial cells, which filled the lumen of glomerular capillaries. A lot of endothelial cells were detached from the glomerular basal membrane and peeled off in the lumens. Extracellular debris in the glomerular capillary lumens probably originated from ruptured endothelial cells. In both cases endothelial cells of the peritubular capillaries were swollen and formed endothelial projections (microvilli) (Figures 3(c) and 2(b)). In the first case (deceased donor exposed to ECMO), small arteries/arterioles showed completely detached and shrunken endothelial cells (Figure 3(d)), whereas in the second case (deceased donor exposed to Takotsubo cardiomyopathy) endothelial cells in arterioles were swollen and only partly detached (Figure 4(d)).

Interestingly, tubular epithelium showed only minimal changes with slightly decreased brush border similar to other 48 cases (changes very likely related to cold ischemia). Desmosomes in tubular epithelium were preserved (Figures 3(a) and 4(c)). Nevertheless, in both described cases serious (post)operative complications followed. To better understand the whole situation, brief history of both cases is presented.

### 2.1. Clinical History from Deceased Donors to Early Graft Loss Outcome

In the first case (Figures 1(b) and 3), the donor of both kidneys experienced cardiac arrest with successful resuscitation; the underlying condition was acute myocardial infarction. Due to cardiorespiratory failure, the donor was supported with ECMO as a life bridge for 4 days. After brain death establishment kidneys were offered for donation. Importantly, the donor had normal urine output and unremarkable markers of kidney function and morphology. There were 3 mismatches (1,1, 1). Recipient (who had no recognized coagulation abnormalities) experienced diffuse subcapsular hemorrhage with multifocal blood leakage through the capsule immediately after completion of the vascular anastomosis of the transplanted kidney. Urgent nephrectomy and implantation of the second kidney of the same donor were performed. Two days after implantation the second kidney transplant was also explanted due to large subcapsular and perirenal hematoma leading to hemorrhagic shock. The patient remained dialysis dependent. No signs of antibody-mediated rejection according to Banff criteria (no glomerulitis or peritubular capillaritis, negative C4d along peritubular capillaries) or thrombotic microangiopathy (no fibrinoid necrosis or fibrin/fibrinogen deposits on immunofluorescence, no typical thrombotic microangiopathy signs on electron microscopy) were found in explanted kidneys.

In the second case (Figures 1(c) and 4) the donor was admitted to intensive care unit due to massive subarachnoid and intracerebral hemorrhage, the diagnoses reported as the cause of death. The donor was also reported to have Takotsubo stress cardiomyopathy presenting with diastolic dysfunction and markedly decreased systolic function. At day 3, brain death was established, and one of the kidneys allocated to recipient in our center. The donor was reported to be hemodynamically stable, with normal urine output and unremarkable markers of kidney function and morphology. The number of HLA mismatches was 1 (0,0, 1). The recipient experienced hemodynamic instability due to subcapsular hematoma, which was recognized few hours after transplantation. Due to primary nonfunction, the recipient needed immediate dialysis. Kidney graft was explanted 3 months after transplantation due to end stage failure.

Transplant kidney biopsy performed 4 days after transplantation revealed glomerular capillary thrombosis and arterial fibrinoid necrosis consistent with acute glomerular and vascular thrombotic microangiopathy, changes not observed in the pretransplant biopsy. Repeated donor specific antibody measurements were negative and there were no other histologic characteristics of antibody-mediated rejection. It was supposed that recipient could have recurrent aHUS, which was confirmed by recipient's complement alternative pathway dysregulation (decreased factor I and C3, elevated urine c5b9).

The time range of cold ischemia storage in 48/50 grafts was 7h21 min-29h25 min, in deceased donor exposed to ECMO the time of cold ischemia was 16h22 min, and in deceased donor exposed to Takotsubo cardiomyopathy it was 9h19 min.

## 3. Potential Risk Factors of Kidney Graft Endothelial Dysfunction in Both Described Cases

It is known that endothelial dysfunction/activation can be associated with numerous factors, for instance, risk factors originated in the donors (such are hypertension and shear stress, age, inflammation, diabetes-associated factors, and atherosclerosis), transplantation procedures (ischemia-reperfusion), or associated with the recipient (immunologic and/or pathophysiologic (miss)match) [11]. However, in deceased donors after brain death (DBD) endothelial dysfunction of kidney transplants due to ischemia and reperfusion, as well as injury associated with time lag from brain death, is almost universal [12].

Therefore, extremely severe and discrepant endothelial injury observed in the two above described kidney graft donors was very unlikely associated with *»*traditional*«* risk factors. Discussion on these topics could, however, be extremely broad and exceed the range of this article. Therefore, we would like to stress out and briefly discuss only factors that clearly stood out from other 48 cases and may be importantly associated with the graft outcome. According to the history of the donors two outstanding clinical peculiarities are noticed: in the first case exposure of the donor to ECMO after massive myocardial infarction with successful resuscitation and in the second case donor's experience with Takotsubo cardiomyopathy.

### 3.1. Extracorporeal Membrane Oxygenation

Extracorporeal membrane oxygenation (ECMO) is used as a standard therapy in critical care for patients with cardiorespiratory failure and nowadays also to manage potential organ donors following cardiopulmonary failure [13, 14]. It is already known that patients on ECMO are at risk of developing acute kidney injury or a systemic inflammatory response syndrome with multiorgan dysfunction [15, 16]. Underlying mechanisms include impaired microcirculatory perfusion [17], gut barrier dysfunction leading to a rise in circulating bacteria products [18], and marked increase in cytokines such as TNF*α* and IL-8, [19] which altogether affect vascular endothelium. The generation of inflammatory mediators results in the widespread activation of the endothelium. Activated endothelial cells in turn increase their expression of adhesion molecules, leading to the increased transmigration of activated neutrophils [15]. It was proposed that neutrophil infiltration (observed also in our case, Figure 1(b)) may be responsible for the end-organ damage associated with ECMO [19].

The outcomes of DBD kidney transplants procured from donors on ECMO have been shown to be comparable to those from non-ECMO donors [20]. However, there are number of possible ECMO-related complications that may have detrimental effect on organs. They are likely dependent on the type of ECMO (i.e., venovenous (pulsatile circulation) or venoarterial (mostly nonpulsatile circulation)) and very likely also on its duration [14]. Importantly, when ECMO is used as a bridge to transplantation, the donor is usually supported by ECMO only for a short time (for instance, in the study of Carter et al. [20] the average ECMO duration was 87 minutes). In contrast, when ECMO is used as a life bridge, patients are exposed to ECMO for days or weeks. According to all above-mentioned risk factors of ECMO, the time of exposure to ECMO is very likely associated with the ECMO-related complications, including endothelial dysfunction. However, to the best of our knowledge, the impact of long-term effect of ECMO on ultrastructure of endothelial cells of donated organs has not been explored yet. Nevertheless, our results suggest that 4 days of ECMO exposure in patient with cardiac arrest may lead to severe endothelial dysfunction.

### 3.2. Takotsubo Stress Cardiomyopathy and Myocardial Stunning

Takotsubo stress cardiomyopathy occurs in the setting of a severe mental or physical stressor, predominantly affecting postmenopausal females, and in the absence of obstructive coronary artery disease. Typically, there is transient reduction in left ventricular systolic function with specific regional wall motion abnormalities [21]. Although the underlying mechanisms are still not known, recent findings indicate that Takotsubo cardiomyopathy can cause chronically impaired peripheral vascular reactivity, including impaired peripheral endothelium-dependent vasodilation, excessive vasoconstriction, and augmented sympathetic activation [22]. The only mechanism known by cardiologists to cause similar transient left ventricular dysfunction as Takotsubo is myocardial stunning [23, 24] which is usually related to transient coronary occlusion and postresuscitation period after the restoration of spontaneous circulation [25].

The results show that while myocardial function has already recovered after acute insult, endothelial cells are more severely impaired than smooth muscle cells, and that this injury persists beyond myocardial stunning. Thus, endothelial-dependent dysfunction can still impair vasodilatation, while ventricular dysfunction is successfully mechanically supported or has already resolved [26]. This foundation refers to coronary endothelium. It would be certainly interesting to explore whether the kidney endothelium responds the same way in such pathophysiologic instances.

## 4. Conclusions

Our observations show that severe endothelial injury of kidney graft microvasculature seen only on ultrastructural level may occur before any significant injury is observed on pathohistological level in preimplantation kidney graft biopsies. These extreme injuries to microvasculature, which are associated with early graft failure, could be promoted by some specific conditions in donor and/or recipient such as long-term ECMO employment, postresuscitation myocardial stunning, or Takotsubo cardiomyopathy. Further studies are needed to determine prognostic significance of severe ultrastructural microvasculature lesions and to evaluate other disease states and conditions that could be associated with severe endothelial dysfunction of kidney allograft.

## Data Availability

The data used to support the findings of this study are included within the article.
